# Pathogenicity and virulence of *Borrelia burgdorferi*

**DOI:** 10.1080/21505594.2023.2265015

**Published:** 2023-10-09

**Authors:** Martin Strnad, Natalie Rudenko, Ryan O.M. Rego

**Affiliations:** a Biology Centre CAS, Institute of Parasitology, České Budějovice, Czech Republic; b Faculty of Science, University of South Bohemia, Branišovská, Czech Republic

**Keywords:** *Borrelia burgdorferi*, Lyme disease, virulence determinants, pathogenicity, clinical manifestations, tick-borne disease

## Abstract

Infection with *Borrelia burgdorferi* often triggers pathophysiologic perturbations that are further augmented by the inflammatory responses of the host, resulting in the severe clinical conditions of Lyme disease. While our apprehension of the spatial and temporal integration of the virulence determinants during the enzootic cycle of *B. burgdorferi* is constantly being improved, there is still much to be discovered. Many of the novel virulence strategies discussed in this review are undetermined. Lyme disease spirochaetes must surmount numerous molecular and mechanical obstacles in order to establish a disseminated infection in a vertebrate host. These barriers include borrelial relocation from the midgut of the feeding tick to its body cavity and further to the salivary glands, deposition to the skin, haematogenous dissemination, extravasation from blood circulation system, evasion of the host immune responses, localization to protective niches, and establishment of local as well as distal infection in multiple tissues and organs. Here, the various well-defined but also possible novel strategies and virulence mechanisms used by *B. burgdorferi* to evade obstacles laid out by the tick vector and usually the mammalian host during colonization and infection are reviewed.

## GRAPHICAL ABSTRACT

**Figure:**
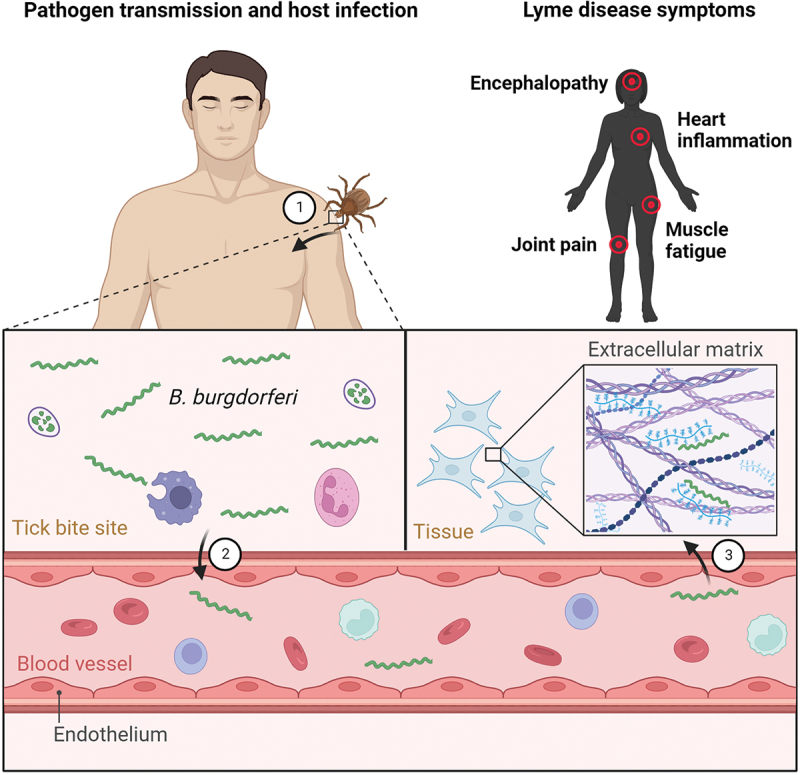

## Introduction

The bacterial pathogen *Borrelia burgdorferi*, also known as *Borreliella burgdorferi*, is the causative agent of Lyme disease (LD) and is transmitted to humans through *Ixodes* ticks. LD is the most common tick infection, with approximately 476 000 cases in the United States [1] and 650 000–850 000 cases in Europe annually [2]. LD occurs in a variety of clinical manifestations, ranging from skin lesions, through musculoskeletal, neurological, to cardiovascular symptoms [3]. The organotrophy associated with *B. burgdorferi* species has an impact on the development of the disease. Although there is already an abundance of knowledge on the causative agent of the disease, an efficacious strategy to reliably overcome LD has not been found. There is currently no vaccine for human use available, although new ways how to cope with the malicious pathogen are being explored [4]. Multiple genes common for *B. burgdorferi* sensu lato species (referred to as *B. burgdorferi* in this Review) display high sequence variability between the species of LD spirochaetes. Genes required for optimal infectivity often differ markedly at both the nucleotide and amino acid level [5,6]. Such variability significantly complicates the design of efficacious immunization strategies including vaccines since the immune responses do not protect against all infectious species to the same extent. This highlights the current strategy which involves clinical development of a multivalent protein vaccine that targets all common serotypes of *B. burgdorferi* [7].

Virulence determinants of *B. burgdorferi* are found both on the linear chromosome and on linear and circular plasmids. Chromosomal virulence genes include *p66, bgp, plzA, rpoN, rpoS*, or *bosR* whereas virulence-associated genes that are present on plasmids encode proteins such as DbpA/B, BBK32, OspC, PncA, VlsE [8]. *B. burgdorferi* possesses a unique, segmented genome comprised of the largest plasmid complement of any characterized bacterium. The plasmids are substantially variable between species and strains, both in number and in sequence [9]. In contrast to a majority of other bacteria, circular plasmids represent a minority of the genome in *B. burgdorferi*. With the exception of the cp-26 plasmid, all circular plasmids are related to each other and are derived from the cp-32 plasmid family. A unique characteristic of linear replicons is that they are terminated by covalently closed hairpin ends [10].

**Figure 1.**
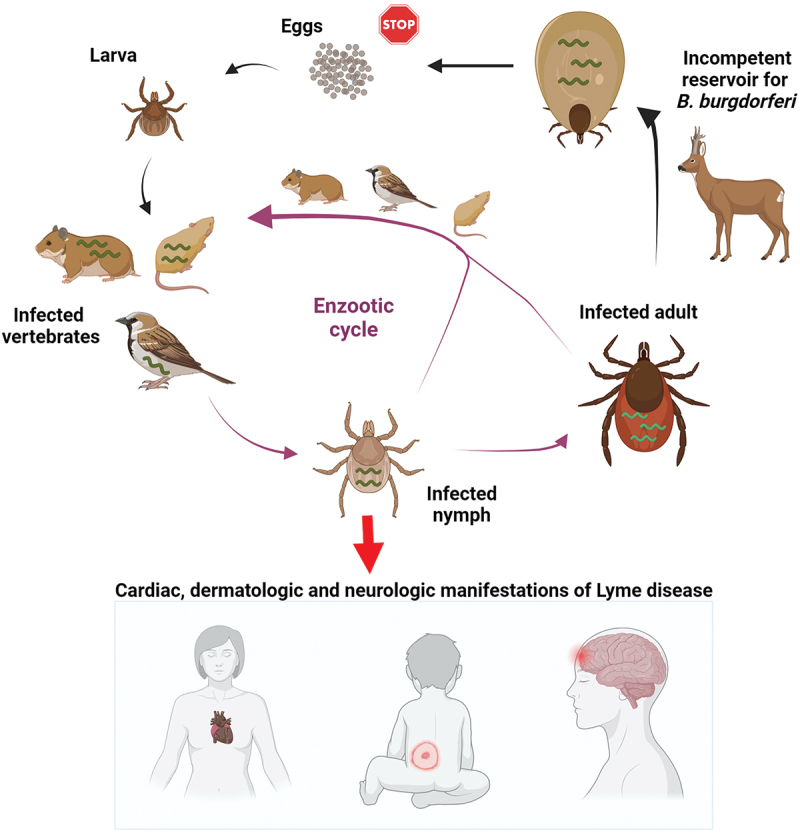
**Enzootic cycle responsible for maintaining** ***B. burgdorferi*** **in tick populations**. *Ixodes* ticks undergo a three-stage life cycle (larva, nymph and adult), with one bloodmeal per stage. Uninfected larval ticks hatch from eggs and feed on a variety of small mammals that can host *B. burgdorferi*. The larva takes an infected bloodmeal and moults into a next tick stage, which is a nymph. Nymphs are responsible for transmitting the majority of infections to humans, leading to variety of clinical manifestations. Deer are important hosts for adult ticks but they are not effective reservoirs for the spirochaetes. Engorged females lay thousands of eggs, however, the spirochaetes are not transmitted through the eggs to the larval progeny.

*B. burgdorferi* exists in an enzootic cycle alternating between a tick vector and a vertebrate host (Figure 1). Acquisition of *B. burgdorferi* by a tick occurs through haematophagous bites of the tick. In order to feed, tick chelicerae cut the host epidermis and the hypostome penetrates through the layers of the skin to secret saliva into the tick bite site on the host and draw blood, possibly, together with *Borrelia* [11]. The spirochaete enters the tick when the larval/nymphal tick takes a bloodmeal on an infected host and colonizes the tick midgut. During subsequent feeding, a handful of spirochaetes colonizing the tick exit the midgut by traversing a peritrophic membrane, a layer of epithelial cells and a basal membrane [12]. The spirochaetes then migrate into the haemocoel as they navigate toward the tick salivary glands, from which they are transmitted to the host dermis via saliva. The salivary route of transmission is highly favourable to the pathogen as it delays the contact with borreliacidal factors present in the host blood. *Borrelia* then migrate through the extracellular matrix (ECM) constituents for haematogenous dissemination into their colonization niches where they cause the damage that can result in development of disease and complete the enzootic cycle [13,14].

To be able to establish an infection and stay inside the host, *B. burgdorferi* must evade the immune pressure exerted by the host’s defence system. The genome sequences of *B. burgdorferi* species revealed that spirochaetes lack the classically defined virulence determinants common to a large number of microbial and, specifically, bacterial pathogens, such as lipopolysaccharides, specialized secretion systems, and toxins [15,16]. *Borrelia* has developed several well-defined mechanisms such as utilization of tick and host proteins, complement evasion, localization into specific niches, dynamic genetic regulation and antigenic variation, in order to survive in the host [8]. There are also several alleged virulence mechanisms not yet unconditionally accepted by the expert community. These include transformations of spiral-shaped replicating *Borrelia* into alternative structural forms such as round bodies or biofilm-like structures [17]. These pleomorphic variants are hypothesized to be able to survive the host immune response or prolonged courses of antibiotic treatment in cases when the subpopulation of *Borrelia* cells switch the phenotype from susceptible to persistent under hostile pressure [18]. Intracellular localization of the spirochaetes, shedding of borrelial outer membrane vesicles, nutritional virulence, modulation of tick microbiota and tick behaviour, and *Borrelia*-induced structural and mechanical transformations of host cells are other potential virulence mechanisms of LD *Borrelia* [19–25].

There are a number of outstanding reviews focusing either on a narrow range of topics in borrelial virulence mechanisms [26–29] or which provide a detailed overview of multiple widely accepted virulence mechanisms [8]. Herein, we guide the reader not only through the complexities of classical but importantly also through the trending virulence mechanisms employed by LD *Borrelia*. In the context of this review, similarly as in the other review of borrelial pathogenic mechanisms [8], the term “virulence determinants” encompasses all genes that impact the infection process, eventhough these factors were often studied and defined in the host animals which are not very susceptible to the disease or show few signs of LD, e.g. mice. This definition includes also genes involved in fundamental metabolic processes necessary for borrelial survival in the host. When the genes associated with a suspected virulence trait are specifically deactivated, it results in a noticeable reduction in infection potential. To sum up, in addition to a better understanding of the bacterial pathogenesis process, identification of virulence factors in the spirochaetal genome may allow the design of new, more efficient and, importantly, more specific intervention strategies than those that are currently employed in combating of LD.

## Diagnosis of Lyme disease

LD has a broad spectrum of symptoms and clinical manifestations. The initial step in distinguishing LD from other illnesses involves the review of all signs and symptoms and gathering subjective and objective data from the patient. If there is potential tick exposure in an endemic area and the individual exhibits an expanding skin lesion called erythema migrans (EM), immediate suspicion for LD should arise. Recognizing EM can be challenging, since EM can be present in various morphological forms, different from the classic bull’s eye [30].

The current reference method utilized for confirmation of physician-diagnosed LD is serological testing. Standard two-tiered testing (STTT) involves an initial enzyme-linked immunoassay (ELISA) as the primary test, followed by supplemental IgM and/or IgG immunoblots. The STTT is recognized for its limited sensitivity in detecting early localized infection, as opposed to very high sensitivity in detecting late infection [31]. Modified two-tier testing (MTTT) is an updated two-tiered testing algorithm, in which confirmation of the infection is done with an ELISA instead of an immunoblot. Multiple studies, both in North America and Europe, have shown that MTTT has improved sensitivity compared with STTT, without losing specificity [32,33]. Some MTTT algorithms have already received FDA clearance for use in serological diagnostics of LD.

The interpretation of serology results can be intricate. It usually takes several days to a few weeks for the immune system to generate a *Borrelia*-specific IgM response, while the development of a *Borrelia*-specific IgG response can take up to two months [34]. Methods to detect IgM antibodies are mainly useful during early disease, since later during infection they often generate false positive signal due to cross-reactions with other infections [35,36]. Another regular weakness of current commercial diagnostic tests is the low sensitivity for less prevalent or rare *Borrelia* species, especially those which exhibit high polymorphism in the commonly detected immunogenic proteins [37,38]. In addition, establishing a clear connection between antibody status and actual infection can be challenging. Non-infected individuals may exhibit immunity long after active LD and test positive, while infected individuals might experience a delay in their antibody response and test negative [39]. Furthermore, specifically in Lyme neuroborreliosis, the antibody production does not always follow the typical immune response of initial IgM secretion followed by secretion of IgG [40].

## Treatment & clinical manifestations of Lyme disease

The course of LD can be divided into three distinct stages – early localized LD, early disseminated LD and late LD. Early localized infection, typically presented as a painless skin rash (EM), is treated with antibiotics. The therapeutic success of LD is dependent on how early is the treatment with antibiotics initiated. A short course of oral antibiotics in most cases efficiently eliminates the pathogen and cures the majority of LD cases. It is recommended that patients with EM are treated with a 10-day course of doxycycline or a 14-day course of amoxycillin or, possibly, cefuroxime rather than longer treatment periods [41]. When LD is diagnosed later, after the spirochaetes have moved from the tick bite site to the secondary sites of infection, the stage is called early disseminated infection. This stage can occur days, weeks, or even months after an infected tick transmits the spirochaetes. Either oral or intravenous antibiotic therapy is recommended for treatment. Clinical manifestations of late LD include a variety of symptoms that are often arthritic and neurologic, and occur months after the transmission of the pathogen [42,43]. Even though the majority of LD cases are cured by antibiotic therapy, around 10–20% of patients experience persistent disease that does not resolve within an extended period of time even after repeated courses of antibiotic therapy [44]. Symptoms such as headache, fatigue, and joint pain are commonly associated with this stage of the infection, which has been defined as post-treatment Lyme disease syndrome (PTLDS) [44]. PTLDS is considered a subset of the term chronic LD, which is an umbrella term with a wide and imprecise definition, often applied to individuals experiencing non-specific symptoms that are believed to stem from an assumed ongoing *B. burgdorferi* infection. These patients may or may not exhibit evidence of past or present LD [45]. Considerable confusion and controversy still exist and questions about the possible existence of this fourth stage of LD, its frequency or the cause of PTLDS is actively discussed by the scientific and medical community. The exact mechanisms and molecular triggers leading to the long-term sequelae of the infection are still undetermined. It is hypothesized that the reduced efficacy of antibiotic therapy may be caused by: a) host-adapted spirochaetes that persist in the tissues or possibly intracellularly inside certain cell types such as fibroblasts and thus are inaccessible to antibiotics [19]; b) excessive inflammatory responses to residual antigenic molecules such as peptidoglycan from killed microbes or autoimmune responses [46]; c) tissue damage after clearance of *Borrelia* [42].

The genetic heterogeneity of *B. burgdorferi* species seems to be the main factor of the diverse clinical manifestations with serious multi-system disorders of human LD. It is not surprising, as out of 22 currently recognized LD spirochaete species, 12 were already detected or isolated from human patients, diagnosed with LD or expected to have a spirochaete infection [47,48]. Within the species that are detected in, or isolated from humans, the frequency of recovery varies greatly. While *B. afzelii, B. bavariensis, B. garinii, B. burgdorferi* sensu stricto (ss) or *B. mayonii* are responsible for the majority of LD cases worldwide, the number of patients in which *B. bissettii, B. kurtenbachii, B. spielmanii* or *B. valaisiana* have been detected is low, and detection of *B. americana, B. lusitaniae* or *B. andersonii* in humans is rare [49, 50]. Diverse clinical manifestations of the disease are connected to different organ tropism that individual LD spirochaete species possess. For example, infection with *B. burgdorferi* ss, the major case of LD in the United States, results in the most common for North America musculoskeletal disorder, Lyme arthritis, that was diagnosed in at least 60% of untreated human patients with EM [51]. In Europe, where *B. afzelii* and *B. garinii* represent the most pathogenic spirochaete species and are recovered from environmental and samples of human origin much more often than *B. burgdorferi* ss, the number of diagnosed cases of arthritis did not exceed 15% [52]. Widely distributed *B. garinii* was confirmed to be related to increased number of neuroborreliosis cases in Europe [53,54]. Occasionally, infections with *B. burgdorferi* ss and *B. afzelii* result in neuroborelliosis as well, however, the number of diagnosed cases is comparably lower. *B. afzelii* predominantly causes skin infections such as EM, acrodermatitis chronica atrophicans (ACA) or borrelial lymphocytoma [55]. Rare cases of ACA were described in patients infected with *B. garinii* as well [56–58]. In addition to the major recognized LD causing species, some other species are occasionally detected in humans as well. While connection of *B. bissettii* [59,60] with cardiovascular disorders was described in patients from Europe [61,62], the status of *B. lusitaniae* [63], and *B. valaisiana* [64] as a human pathogen still needs a valid confirmation [65]. Tick co-infection with other pathogens such as *Rickettsia* and *A*. *phagocytophilum* does not significantly increase in clinical symptoms or disease duration [66].

## Vaccine development

Although there are currently no approved vaccines against LD for human use, multiple studies have built a solid foundation for further vaccine development. *B. burgdorferi* produces multiple antigenic and immunologically accessible molecules which were shown to be promising vaccine candidates but which have never reached the market. These molecules include mostly the surface-exposed borrelial proteins such as BBA52, BB0405, BBI39, DbpA, BBK32, OspC, with data showing that combination vaccines composed of DbpA, BBK32, and OspC are more powerful than single formulation or double component cocktails [67–73].

The only licenced vaccine to prevent LD was LYMERix, with efficacy of nearly 80% [74]. After being available on the market few years, the vaccine was pulled from the US market in 2002 due to numerous reports of severe side effects. There was a hypothesis suggesting that the vaccine antigen, outer surface protein A (OspA), acted as an autoantigen, potentially causing arthritis. However, the adverse effects were never definitively substantiated [75]. The Valneva-Pfizer “VLA15” is currently the only LD vaccine candidate in advanced clinical development (phase 3). This multivalent protein subunit vaccine targets OspA and provides protection against the six prevalent OspA serotypes expressed by the *B. burgdorferi* species commonly found in North America and Europe [7,76].

In order to address the considerable variability observed in proteins across different strains and species of *B. burgdorferi*, effort has been put on creating a multivalent chimeric vaccines that offer protective effects against various species of LD, aiming to tackle the heterogeneity inherent in the pathogen [70,77,78]. Multivalent OspA-based formulations are currently common targets in vaccine development. To create self-assembling nanoparticles, OspA was combined with bacterial ferritin. These OspA-ferritin nanoparticles induced robust and long-lasting antibody responses against the major serotypes in both mice and non-human primates [79]. To achieve broad protection with a single recombinant antigen, a strategy called grafting, or epitope reshaping, has been successfully tested for production of neutralizing antibodies against the six clinically most relevant OspA serotypes [80]. Besides vaccines based on recombinant protein technology, DNA tattoo and lipid nanoparticle-encapsulated DNA vaccination represents a promising strategy for prevention of LD [81–83]. DNA vaccines present several potential benefits compared to conventional strategies, such as the activation of both B- and T-cell responses, enhanced vaccine stability, the absence of any infectious agents, and the straightforward large-scale production process [84].

## Methods used to identify virulence mechanisms and determinants

### Genetic modifications

Virulence is defined by the ability of a microorganism to cause disease in the host. Modern strategies that are being used to uncover the fine details of bacterial virulence mechanisms encompass a combination of biochemical, genetic, imaging, and computational techniques. The currently used genetic tools for manipulating *B. burgdorferi* are sufficiently mastered for precise and effective genetic investigations and were recently reviewed in great detail by Rosa and Jewett [85]. Targeted genes can be selectively rendered inactive and the resulting mutant phenotype can be examined for infectivity in the experimental mouse-tick transmission cycle, thus elucidating the actual functions of borrelial virulence factors during infection of the host. Genetic manipulation of *B. burgdorferi* is typically achieved via allelic exchange. The transformation protocol often makes use of electroporation for transfer of foreign DNA [86]. The selection of a mutant with either gene disruption or gene complementation is achieved by using a selectable antibiotic marker. Suicide vectors are commonly utilized for targeted gene inactivation, as the mutant is generated by allelic replacement of the wild-type gene with a disrupted copy [85,87]. The functional complementation of the mutant is typically achieved through shuttle and suicide vectors for *trans* and *cis* complementation, respectively [85].

Transposon mutagenesis and signature tagged mutagenesis (STM) are more recent genetic approaches that were developed to identify novel bacterial virulence factors and were already exploited in borrelial research [88–90]. Both methods represent a technical advance over other random mutagenesis approaches such as radiation and chemical mutangenesis [91] as they utilize transposable genetic elements to randomly inactivate multiple genes, with each specific mutation occurring in an individual clone. A Himar1-based transposon suicide vector is often used for the delivery of the gene insertions [88,92]. The principle of STM is based on molecular barcoding with unique DNA sequences (signature tags), which allow mutants to be differentiated from each other. This allows high-throughput screens of large number of different mutants within a single animal. By comparing the signature tags of the input pool of mutants, i.e. those which were inoculated into an animal, and the recovered pool of mutants, i.e. those which survived in the animal, the important virulence factors can be identified.

### Gene expression analysis

DNA microarrays have been used to study changes in the global expression profile of *B. burgdorferi* grown under various *in vitro* and *in vivo* conditions [93,94]. For mimicking the growth conditions in the mammalian host, a dialysis membrane chamber implanted into the rat peritonea is considered a top-notch model as it allows to obtain high number of host-adapted spirochaetes for subsequent analysis [95,96]. By comparison of expression profiles of spirochaetes grown under fed-tick, unfed-tick, and mammalian growth conditions, it was shown that mostly the extracellular lipoproteins are dynamically regulated during borrelial transitions between the tick vector and the host, hinting to their important role in borrelial pathogenesis [93]. The common drawback of the DNA microarray technology is that only the annotated coding regions of the genome can be profiled. To meet the limitations of probe performance in DNA microarrays, RNA sequencing (RNA-Seq) has been used to explore for instance the antisense transcripts and noncoding/small RNAs [97,98].

The *in vivo* expression technology (IVET) is a genetic tool that allows selection of genes specifically expressed *in vivo* in complex environments. In contrast to DNA microarrays technology and RNA-Seq, IVET does not require borrelial RNA isolation for the transcriptomic investigation and provides purely qualitative data with no information about the quantity of studied gene product. This promoter-trapping technique provides means for genome-wide identification of the borrelial genes that are expressed for instance during murine infection. Using IVET for the first time in *B. burgdorferi*, an RpoS-independent gene *bbk46* was identified and shown to be important for evasion of the host adaptive immunity [99]. Using an IVET reporter cassette containing antibiotic resistance *aacC1* gene and borrelial *pncA*, Casselli and Bankhead [100] identified a number of constitutively expressed promoters, *in vitro*-induced promoters, and *in vivo*-induced promoters in *B. burgdorferi*.

### Imaging techniques

The rapid development of imaging technologies has revolutionized our ability to visualize the delicate intricacies of *Borrelia*-host interactions at very different scales. Imaging methods and applications encompass a palette of options from which one might select, spanning from whole animal imaging techniques to modalities that are able to reach subatomic resolution [101,102]. Bioluminescent imaging has enabled one to follow the infection of *Borrelia* in an entire model animal over time by non-invasive means in natural environment [101]. Another sophisticated technique that has allowed the investigation of *Borrelia* pathogenesis, including dissemination and invasion of the host, is intravital microscopy [14]. Intravital imaging allows real-time characterization of a pathogen in a living host at specific locations. For intravital imaging, spinning disk confocal microscopy (SDCM) and two-photon microscopy (2PM) are often employed [14,103–105]. The spinning disk in the SDCM setup allows for imaging of very fast dynamic processes in live specimens and reduces phototoxicity. With improved laser penetration by low energy photons and significantly reduced phototoxicity, 2 PM is very convenient for real-time intravital imaging of *Borrelia* in the mouse dermis [104,105]. Using SDCM in an intravital setting, the details of vascular extravasation of *B. burgdorferi* were visualized, showing that the bacterium engages in multi-stage interactions with the endothelial cells, including tethering and dragging interactions, and stationary adhesion to the vasculature [14]. Intravital imaging is typically accomplished by using fluorescent labelling of the pathogen, preferably by expression of a fluorescent fusion protein such as GFP. To avoid the potential issues with bulkiness of the GFP, new small fluorescent probes for investigation of spirochaete morphology and motility are being explored [106].

Light and electron microscopy are usually used to provide a cellular-level understanding of mechanisms of *Borrelia* infection (spirochaete-host cell interactions) [22,107]. To address the structure-functional contributions of borrelial proteins to bacterial virulence and disease pathogenesis, modern high-resolution imaging modalities can be utilized. Atomic force microscopy-based techniques enable the study of protein–protein interactions at the single-molecule level, for instance, in uncovering the important interactions that facilitate the spirochaete to efficiently translocate in the extracellular matrix of a host [13]. Nuclear magnetic resonance-based techniques can go even deeper with resolution power and determine the location of the binding site and the dynamic structural rearrangements that occur upon binding, with amino acid precision [102,108].

## *Borrelia*-specific versus common virulence factors

Virulence factors can be classified as pathogen-specific (no orthologous protein in other bacteria) and common (one or more orthologous proteins in different pathogenic bacteria) based on whether the virulence determinant is present in a single species or it is found in multiple different organisms. *B. burgdorferi* contains a high number of borrelia-specific virulence factors such as OspC, OspA, VlsE but also contains several virulence determinants that can be found in other microorganisms. For instance, *B. burgdorferi* utilizes non-toxin adenylate cyclase *cyaB*, an enzyme that produces secondary messenger cyclic adenosine monophosphate (cAMP), to modify borrelial gene expression and protein production in order to enhance its virulence in the vertebrate host [109]. CyaB ortholog can be found in other pathogenic bacteria such as *Pseudomonas aeruginosa* or *Vibrio vulnificus* [110,111]. Cyclic dimeric guanosine monophosphate (c-di-GMP) is an ubiquitous second messenger found in many prokaryotes such as *Mycobacterium tuberculosis, Vibrio cholerae*, or *Salmonella enterica* that regulates a wide span of physiological processes including bacterial protein secretion, multicellular behaviour, virulence, motility, and cell development and differentiation [112,113]. Although c-di-GMP has various downstream effectors in bacteria [114], PlzA protein is the only universal c-di-GMP binding protein in *B. burgdorferi*. The regulatory processes of c-di-GMP are mediated through binding with the PilZ domain, the C-terminal part of PlzA [115]. In *B. burgdorferi*, c-di-GMP regulates the adaptation processes to the tick and mammalian environment via the interaction with PlzA [116]. PilZ domains are found in multiple bacterial taxa such as actinobacteria, proteobacteria, or spirochaetes [112].

LuxS is another virulence factor found in *Borrelia* [117] as well as many other pathogens such as *Bacillus anthracis* [118], *Staphylococcus epidermidis* [119], *or Haemophilus parasuis* [120]. In addition to the mostly referred genes, such as *ospA, ospB, ospC, crasp(s), erp(s), vls, dbpA/B, flaB, p66, rpo(s)*, the group of genes involved in sensing and transduction of environmental signals deserves more attention. To differentially synthesize proteins during the infectious cycle, *Borrelia* is equipped with regulatory networks to sense its environmental signals. Quorum sensing is a mechanism by which many prokaryotes monitor their surroundings by producing and responding to signalling molecules known as autoinducers [121]. The genome of *B. burgdorferi* encodes for a number of enzymes involved in quorum sensing, such as MetK, LuxS, and Pfs [15] that enables the spirochaete to synthesize an autoinducer type 2 (AI-2) mediating in this way quorum sensing that can function in both the mammalian host and the tick vector. Therefore, it is possible to regulate the differential expression of *Borrelia* genes in different environments [117,122]. The bacterium has to interact with different tissues during its life cycle, derive nutrition mostly from warm-blooded mammalian hosts as well as from the tick vector, and avoid clearance by host and vector immune responses. By exploiting quorum sensing, a whole borrelial population can synchronize production of molecules required for infection and survival. There are additional advantages in having the whole population of *Borrelia* to coordinate particular functions. For example, simultaneous transmission of large numbers of spirochaetes from a tick to a warm-blooded host might improve the odds that at least some spirochaetes survive the host’s immune responses and establish disseminated infection [122]. The quorum sensing mediated by LuxS is considered as the main system used for cell-to-cell communication. Although an earlier study suggested that *luxS* is redundant for *B. burgdorferi* tick colonization, transmission to a mammal, or establishment of infection [123], a more recent study demonstrated that the *luxS* mutant was markedly less infectious than the wild type and that *luxS* gives a fundamental advantage to the spirochaete during vertebrate infection [124].

## Tick transmission factors of *B. burgdorferi*

*B. burgdorferi* naturally persists in an enzootic cycle that primarily involves *Ixodes* ticks and mammals. Adult and nymphal ticks are the most important stages in the transmission of the bacterium. *B. burgdorferi* is not propagated from the adult female tick to their offspring, or it happens only rarely [125,126]. The transovarial transfer of *B. burgdorferi* has been occasionally documented in the literature, but these records may probably stem from confusion between LD species and relapsing fever (RF) *Borrelia* [44]. Nymphs are the most harmful tick stage for humans, because the risk of an infection in the majority of cases comes from the bite of this developmental stage of the tick vector. Tick larvae are very rarely infected and the adult ticks are large enough to be noticed early and removed appropriately. *Ixodes* ticks take only one bloodmeal per life stage (larva, nymph, adult) after hatching from eggs. At least seven tick-borne zoonoses are transmitted by members of the *Ixodes* complex in the northern hemisphere, and include diseases such as LD, ehrlichiosis, babesiosis, anaplasmosis, tick-borne encephalitis, Rocky Mountain spotted fever, Powassan encephalitis disease, etc.

*B.* *burgdorferi* species are host-propagated pathogens that shuttle between tick vectors and vertebrate hosts. *B. burgdorferi* in the unfed ticks is predominantly located in the midgut lumen, where the spirochaetes have to avoid the tick innate immune responses, manifested especially in the form of antimicrobial peptides (AMPs) [127,128]. Further down the road primarily in the tick haemocoel, the bacteria have to avoid the haemocyte-mediated processes (phagocytosis, encapsulation and nodulation) as well as damage mediated by AMPs, lysozyme, complement-like factors, and reactive oxygen and nitrogen species [129–132]. *Borrelia* multiply in the midgut to many thousands of cells [133]. Spirochaetes reside in the midgut during tick starvation phases as well as tick moults. Upon blood feeding, *Borrelia* escape from the gut to navigate toward the salivary glands and onwards into a new host [134]. An alternative transmission pathway, in which the tick salivary glands are considered unessential for successful transmission, might be exploited by some of the major European species. In the study by Pospisilova et al. [135], the authors show that *B. afzelii* did not reach the tick salivary glands at any stage of feeding and that tick saliva is redundant for infectivity of this particular species. The possible differences in the transmission mechanisms in *B. burgdorferi* genospecies were further strengthened by a study demonstrating no presence of *B. afzelii* in the salivary glands of unfed ticks [136]. On the contrary, in the same study, other European species *B. lusitaniae, B. spielmanii* and *B. garinii* were detected in both midgut and salivary glands suggesting that the migration of these spirochaetes between midgut and salivary glands might not be activated by the bloodmeal [136].

### Protein interactions between LD *Borrelia* and ticks

**Figure 2.**
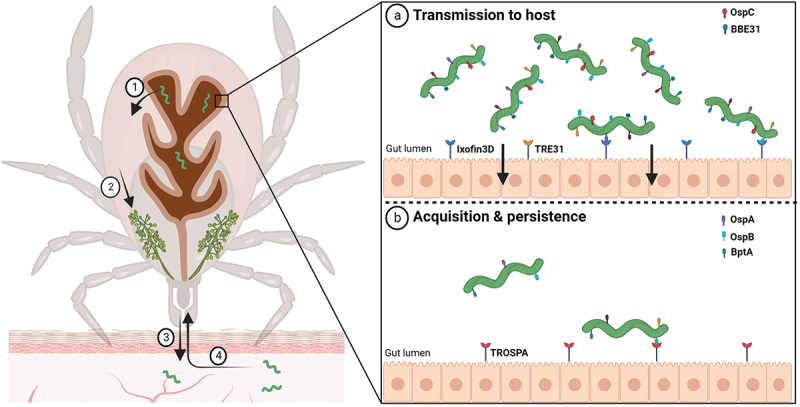
**Molecular interactions at the tick midgut luminal interface involved in transmission and acquisition/persistence of *B. burgdorferi* in ticks**. When an infected tick starts feeding on the host, spirochaetes present in the tick midgut multiply, traverse the gut barrier to reach the haemocoel (1), navigate toward the tick salivary glands (2), and ultimately infect the host (3). (a) upon blood intake, the spirochete changes its protein coat to produce molecules such as OspC and BBE31, which enable the pathogen to escape from gut and invade the tick salivary glands. Tick epithelial cells express molecules Ixofin3D and TRE31, which facilitate the process of gut penetration. (b) *Borrelia* cells ingested in the bloodmeal bind to the tick gut and stay there until a next tick feeding. *B. burgdorferi* expresses several-exposed lipoproteins such as OspA, OspB and BptA, which protect spirochaetes from bactericidal components present in the host blood and enable them to colonize the tick gut. OspA interacts specifically with the tick receptor TROSPA, thereby enabling efficient colonization of the vector.

*Borrelia* adapts to the transition between the tick vector and vertebrate host by preferential gene expression. To optimize colonization and persistence in the tick midgut, *Borrelia* has evolved complex strategies, how to exploit the tick midgut milieu (Figure 2). The successful tick colonization depends on and is governed by complex molecular interplay of spirochaetes with the gut epithelium receptors. The temporal pattern of *ospA* (outer surface protein A) gene expression together with a number of functional studies [137,138] indicate that the abundantly expressed OspA plays a vital role in spirochaete persistence in the vector. OspA-deficient spirochaetes are unable to colonize the tick since they fail to bind to the tick gut [137]. The impaired attachment of *B. burgdorferi* to the tick gut effectively obstructs all subsequent downstream transmission events. Tick receptor for OspA (TROSPA), located on the luminal surface of gut epithelium, interacts with the spirochaete OspA lipoprotein and facilitates the tick colonization. TROSPA interacts specifically with OspA and was shown to be upregulated when spirochaetes are ingested [139]. RNA interference of TROSPA prevents efficient colonization of the tick and reduces borrelia transmission to the host [139]. Similarly, borrelial outer surface lipoprotein BBE31 binds to the vector molecule TRE31 located in the tick gut [140]. *Borrelia* infection induces TRE31 expression and silencing of TRE31 results in a significant decrease of the borrelial loads in the tick haemolymph and salivary glands [140].

Outer surface protein B (OspB) is another surface-exposed lipoprotein essential for adherence, survival, and persistent infection of the tick midgut [137]. OspB-deficient microbes are capable of migrating to the feeding ticks together with the bloodmeal but their ability to adhere to the tick gut and survive within the arthropod vector is significantly diminished [141]. While the binding partners for BBE31 and OspA are known, the mechanism how OspB exerts its function is still unclear. Studies have also identified borrelial proteins required for persistence inside the tick, which are critical for survival during long off-host periods between bloodmeals. BptA (bbe16), a lp25-encoded surface-exposed lipoprotein, was found to be essential for this persistence of *Borrelia* in ticks but the molecular mechanisms by which BptA promotes the persistence remains to be elucidated [142]. DksA is involved in the transcriptional response to nutrient limitation and supports the transcription and translation of genes contributing to *B. burgdorferi* infectivity such as of virulence-associated lipoprotein OspC [143,144]. Another potentially important factor in the tick vector stage of *B. burgdorferi* is an outer membrane protein BBA52. Tick acquisition of mutants lacking functional *bba52* was shown to be reduced [145]. Using a yeast surface display library, Ixofin3D and *I. scapularis* dystroglycan-like protein (ISDLP) were identified as other candidate tick gut binding partners of *B. burgdorferi* [146,147]. The expression of both Ixofin3D and ISDLP is increased in *Borrelia*-infected tick gut during bloodmeal ingestion. Ixofin3D was suggested to facilitate borrelial congregation to the tick gut that ultimately helps *Borrelia* to navigate and escape from the gut [146].

The migration of the spirochaetes toward the tick salivary glands is accompanied by downregulation of tick gut specific borrelial proteins such as OspA and concomitant upregulation of proteins such as OspC that helps *Borrelia* to reach the tick salivary glands [148] and infect and colonize the host [149]. The *I. scapularis* salivary protein Salp15 binds specifically to OspC, thereby enabling the bacterium to avoid clearance mediated by host anitbodies [150]. The same function was demonstrated for the homologue of Salp15 from *I. ricinus* Iric1. In addition, Iric2 and Iric3 from *I. ricinus* are capable of OspC binding with high affinity [151]. Although *Borrelia* evolved its own mechanisms to combat immune defence of the host, the transmission via tick saliva gives spirochaetes certain adaptive benefits. Salivary gland products also provide the first line of defence for *Borrelia* against the host innate immune system. Tick salivary proteins play a role in the inhibition of the host immune and homoeostatic responses that are raised against them. This includes the suppression of activation of phagocytic dendritic cells and macrophages, including the inhibition of natural killer cells that are responsible for chemokine and cytokine production, and disabling of granulocyte recruitment to the tick bite site [152].

**Figure 3.**
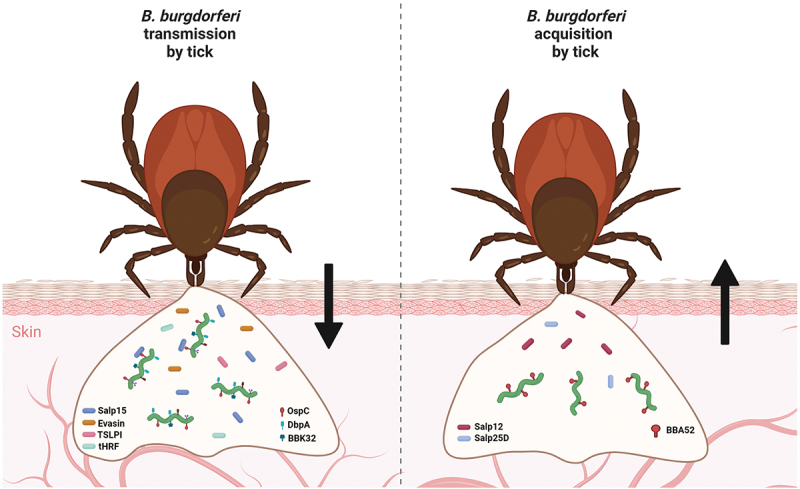
**Molecular factors involved in transmission and acquisition of *B. burgdorferi* by ticks**. numerous molecules produced by both a tick vector and *Borrelia* are required for successful early-stage infection of the host and tick acquisition of *B. burgdorferi*. Many tick proteins (Salp15, evasins, TSLPI, tHRF, etc.) and borrelial proteins (e.g. OspC, DbpA, DbpB, BBK32) are known to be necessary for transmission. Chemotactic tick protein Salp12 and antioxidant Salp25D, together with borrelial BBA52, were shown to be critical for host-to-tick acquisition of *Borrelia*.

When an uninfected tick feeds on an infected host, the spirochaetes are directed toward the tick bite site by specific environmental and chemotactic stimuli. Tick protein Salp12 is released into the skin tissue as a component of the tick saliva, where it is sensed by resident *Borrelia* [153]. This recognition contributes to attraction and relocation of the spirochaetes into the tick feeding pit. Salp12 is expressed and present in the salivary glands as well as midgut. It was shown that during tick feeding, the expression of Salp12 in midgut of the tick was significantly higher than Salp12 expression in the salivary glands at various investigated time points, possibly creating a concentration gradient which directs the bacteria toward the tick midgut. Passive immunization and disruption of Salp12 in the tick salivary glands were shown to decrease acquisition of the spirochaetes by the vector [153]. Using similar experimental procedure, it was shown that the tick antioxidant protein Salp25D also play a fundamental role in the acquisition of *B. burgdorferi* [154] (Figure 3).

## Pathogenic mechanisms at the host–pathogen interface

Genome sequencing revealed that *B. burgdorferi* lacks virulence factors common to many microbial and bacterial pathogens, such as specialized secretion systems, toxins, or extracellular proteases with the exception of HtrA [15,155]. As a notorious extracellular pathogen, the spirochaete most likely does not damage the host cells by penetrating them or multiplying in them. Neither does *Borrelia* produce toxins that would impose direct damage to the host cells membranes, kill the host immune cells, or inhibit protein synthesis. The outer membrane of *B. burgdorferi* is structurally distinctive among bacterial cell envelopes and it is a common target of host immunity [156]. Unlike typical diderms, *B. burgdorferi* does not produce the endotoxin lipopolysaccharides but secrete extensive surface lipoproteins at the host–pathogen interface [15]. These outer membrane lipoproteins play an essential in the borrelial virulence by suppressing immune responses of the host and by mediating interactions with the targets tissues [157]. Despite the activation of both the innate and adaptive immune systems, *Borrelia* is often able to survive in the hostile environment to cause infection, which then can become persistent. LD has not been associated with severe inflammatory over-reaction such as cytokine storms known from cytomegalovirus, Epstein-Barr virus, streptococcus, and many other microbes. *Borrelia* is better known for its immune-evasion mechanisms, expression of immunomodulatory surface proteins, downregulation of immunogenic proteins, and antigenic variation, which help to elude immune responses of the host [158].

### Evasion of host innate immunity

The mammalian host immune response to infection with LD spirochaetes begins with effector components of the innate immune system. Nitric oxide (NO), generated in multiple biologic tissues and cells by specific nitric oxide synthases, is a known player in innate immunity responses against bacterial pathogens [159]. Although NO is a powerful antimicrobial agent and its production is elevated during borrelial infection [160], *B. burgdorferi* is not significantly affected by NO during mouse infection [161]. In contrary to elevated levels of NO during infection, the spirochaete is able to suppress the production of additional common toxic molecules such as reactive oxygen species (ROS) via the mTOR pathway in order to avoid killing [162,163]. *B. burgdorferi* produces multiple proteins which were implicated to ameliorate the effects of ROS exposure, including BmtA, SodA [164,165]. Recently, a gene cluster *bb_0554-bb_0556* (*xdhACB*) encoding a xanthine dehygrogenase and transmembrane proteins BB0017, BB0164, and BB0202 were shown to play a significant role in ROS resistance [166,167]. In addition, the capacity of lysozyme, an antimicrobial enzyme and a major constituent of innate immunity, to kill *B. burgdorferi* is quite limited [168].

### Complement evasion by LD *Borrelia*

The complement system is considered a functional bridge between innate and adaptive immune responses and is known to be activated via three pathways – the classical pathway, lectin pathway, and alternative pathway. Upon activation by the pathogen, the complement system opsonizes bacterial surfaces and induce a series of proinflammatory responses that ultimately lead to bacteriolysis [169]. Vertebrate host range for *Borrelia* is determined by spirochaetes sensitivity to complement of particular animal species; the ability to bind factor H (FH) and factor-H-like protein 1 (FHL-1) appears to depend on the *Borrelia* genotype [170, 171]. *B. burgdorferi* species vary in their susceptibility to complement. *B. afzelii* and *B. burgdorferi* ss are strongly serum (complement) resistant while *B. garinii* is more sensitive to most mammalian serum [172,173]. The spirochaete has evolved mechanisms to evade destruction from the complement system by producing a set of outer surface lipoproteins that interact with host complement molecules and manipulate the native activities of the defence system [174]. *Borrelia* can either bind directly to complement components or the spirochaete can interact with the host-derived regulators of the complement system in order to attenuate complement activation.

Direct interference is mediated by a spectrum of outer surface lipoproteins such as BBK32, OspC, and BBA70. BBK32 specifically inhibits the classical pathway through high-affinity binding of the C1r subcomponent of C1, entrapping C1 in its zymogen form [175]. OspC was shown to bind C4b in both *B. garinii* and *B. burgdorferi* ss, blocking formation of C3 convertase and thereby restricting killing via the classical and lectin pathways [176]. OspC, together with other surface molecules such as OspA, CspA, Erp-family proteins, and BBA70, are capable of binding plasminogen, which is a known complement inhibitor [177–182]. Indirect strategies include the production of complement-regulator acquiring surface proteins (CRASPs) which encompass CspA, CspZ, and OspE paralogs to inhibit the complement responses. These molecules attenuate complement activation on the borrelial surface by binding to complement regulator FH or FH-related protein 1 (FHR-1) [183,184]. CspA (CRASP-1) attenuates complement on two central activation levels, C3b generation and assembly of the terminal complement membrane attack complex [178,185]. European species *B. bavariensis* produces surface proteins BGA66 and BGA71 similar to CspA. Both molecules bind complement components C7, C8 and C9, and prevent assembly of the terminal complement complex [186]. CspZ (or CRASP-2), a second FH/FHL-1-binding protein, also downregulates the formation of C3 and C5 convertases on the spirochaete surface. A third type of FH-binding proteins are OspE paralogs ErpP/ErpC/ErpA (also known as CRASP-3, CRASP-4 and CRASP-5). The outer surface protein OspE binds to Factor H as well as various members of complement factor H-related (CFHR) proteins [187]. ErpA and ErpP, the OspE-related proteins, bind the complement factor H-related proteins CFHR1, CFHR2, and CFHR5, while ErpC binds only CFHR1 and CFHR2 [174,188].

### Antigenic diversity and variation in LD *Borrelia*

After activation of adaptive immunity, antigen presenting cells utilize bacterial peptides and present them to B and T cells. T cells release cytokines and stimulate macrophage activation while B cells produce specific antibodies that bind borrelial outer membrane epitopes. The escape from adaptive immune responses is mediated primarly by the VlsE antigenic variation system [189]. Antigenic diversity can fundamentally extend the time a pathogen maintains an infection within a host and avoids eradication by the host immune system. The initial set of surface-presented antigenic molecules stimulate an immune response against the dominant antigens. If the pathogen changes the composition of its antigenic coat to new variants, the microbe escapes the immune response and continues infection until the host generates a new response against the latest variants [190]. LD *Borrelia* species are well known for differential gene expression and alterations in antigenic structure during their life cycle. A prototypical example is downregulation of OspA lipoprotein during transmission while the expression of OspC is upregulated. Subsequently, OspC, which is critical for early stage of mammalian infection is downregulated and *Borrelia* produces genetic variants at the *vls* (variable membrane protein-like sequences) locus to enable long-term infection of the host [149,191].

OspC is a dominant highly polymorphic antigen of LD bacteria, that is heavily targeted by the host immune system. *Borrelia ospC* is more diverse than any other studied gene to date; it is known to be able to establish the secondary immune response or immune memory in hosts [192], association between *ospC* genotypes and invasiveness in human patients and infected animals have been reported in multiple studies [193–195]. The gene encoding for OspC is mapped to cp26, a 26-kb circular plasmid that is a stable component of the segmented *B. burgdorferi* genome [196]. OspC is transiently but absolutely required during the early stage of infection and neither *vlsE* nor *ospA* can compensate for the absence of OspC and restore infectivity to an *ospC* mutant [191]. OspC is a protective antigen [197], due to its high sequence variability, protection is generally strain specific [198]. Multiple alleles are circulating among reservoir hosts and tick vectors [192]. *OspC* alleles A, B, and L were detected in Europe and North America in vectors and hosts including humans. Six *ospC* alleles are prevalent in Europe and four of them were detected in human samples. Ten *ospC* alleles were identified in the western United States. Four *ospC* alleles were abundant in the southeastern United States [199]. OspC has also been suggested to play a role in host selectivity [192], plasminogen binding in hosts [200], defining *Borrelia* invasiveness in rodents [200], dissemination during mammalian infection [201], salivary gland migration in the tick [148], evasion of innate immunity [202], binding a tick salivary protein that inhibits complement [150,203], conferring bloodstream survival [204], and contributing to *Borrelia* strain-specific differences in tissue tropism [205]. OspC suppresses the classical and lectin complement pathways and competes with complement protein C2 for C4b binding [176]. OspC is important for *Borrelia* in macrophage phagocytosis, reducing the uptake of the bacterium by human and murine macrophages [206].

Decorin binding proteins A and B (DbpA/B), that are important for motility, bind primarily to the connective tissue proteoglycan decorin and facilitate host colonization by the spirochaete [13]. Although not absolutely essential for infection, the important role of these adhesins for the overall virulence of *B. burgdorferi* was demonstrated in a number of studies [67,207,208]. DbpA and DbpB deletion mutants display marked attenuation in mammals, but particularly early during the course of infection [209,210]. Recovery of mutant bacteria from tissues distant to the inoculation site is diminished as well [208,210]. Besides binding to specific glycosaminoglycan (GAG) ligands in order to anchor the spirochaete in the destination niches, DbpA and DbpB help in host colonization by enhancing borrelial translational motility in the low shear stress environments [13]. DbpA sequence variability among different species of *B. burgdorferi* is high, resulting in pronounced differences in their GAG affinities [6,211,212]. It was shown that allelic variations of *B. burgdorferi* DbpA affect tissue tropism and disease manifestation of different LD genospecies [213].

Besides temporal changes in the antigenic coat as a result of tick vector-host circulation and high sequence variability between borrelial genospecies, *Borrelia* are capable of *in situ* antigenic variation to avoid detection from host adaptive immune response. Recombinational switching of a gene locus, or the process of alteration of the pathogen’s surface antigens in order to avoid detection from host adaptive immune response, is one of the most effective attributes of immune evasion by human pathogens. Concomitant with the development of the host acquired immune response and OspC downregulation, robust synthesis of VlsE is initiated. In *B. burgdorferi* strain B31, the *vlsE* gene is encoded on the 28-kb linear plasmid, lp28–1, less than 100 bp from the right telomere. VlsE is dissimilar to OspC, OspA, or Dbps, as it is abundantly present on the spirochaetes surface during persistent infection [214, 215]. The silent cassettes at the *vls* locus vary during the course of infection as well as between species and within species [216–218]. The VlsE antigenic variation system contains the *vlsE* expression site and 15 silent cassettes, thus providing multiple possible recombination events for producing a variety of VlsE epitopes through unidirectional, segmental gene conversion [189,219]. The *vlsE* cassette region can have over 90% nucleotide sequence identity with the *vls* cassettes. European species *B. garinii* and *B. afzelii* were shown to have less *vls* silent cassettes than *B. burgdorferi* B31. *B. garinii* strain Ip90 carries 11 *vls* silent cassettes and *B. afzelii* strain ACAI encodes 14 *vls* silent cassettes [220]. The cassettes can recombine in seemingly random manner (although there is some preference for certain silent cassettes), ranging from single nucleotide substitutions to almost full replacement of the *vlsE* site, with switch rate of approximately 0.033 per cell generation [221]. The RuvAB helicase complex apparently facilitates the *vlsE* recombination [222]. There are two types of sequence changes; templated and non-templated. The templated changes are those which correspond to sequences in the *vls* silent cassettes, whereas non-templated changes are often represented by point mutations. The degree of recombination events and switch length of nucleotides are influenced by the immune pressure. The spirochaetes in wild-type immunocompetent mice display longer stretches of recombination and average switching rate than in immunodeficient mice [221]. The antigenic variability of VlsE facilitates another novel escape mechanism from innate immunity described as epitope shielding [223]. The VlsE protein can effectively block the binding of antibodies which target immunogenic borrelial proteins such as arthritis-related protein Arp.

### Motility-driven pathogenesis

**Figure 4.**
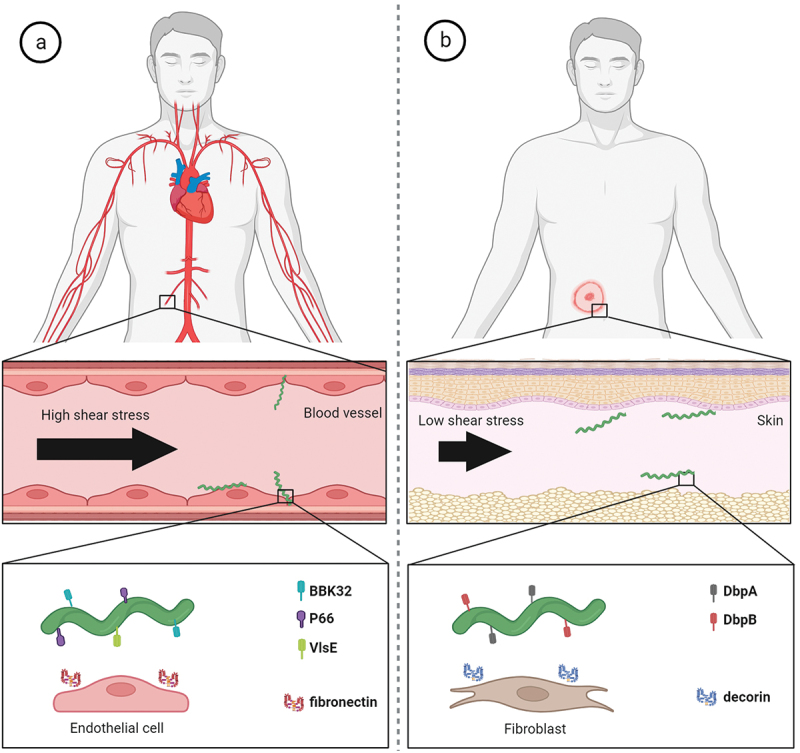
**Molecular factors directly involved in active migration and dissemination of *Borrelia* in humans**. (a) *B. burgdorferi* has to overcome high shear forces generated by blood flow in order to adhere to vascular surfaces for extravasation. The interactions of borrelial protein BBK32 with vascular fibronectin allow active migration in high shear stress environment and borrelial proteins P66 and VlsE aid in vascular transmigration. (b) in low shear stress niches such as extracellular matrix of target skin tissue, borrelial surface-exposed proteins DbpA and DbpB facilitate the translational motion by interactions with matrix molecules such as decorin.

*B. burgdorferi* possesses surface adhesins that are well adapted to aid in its dissemination and colonization strategies (Figure 4). During its infectious cycle, *B. burgdorferi* needs to cope with high blood flow-mediated shear stress in the host vasculature. BBK32 and P66 borrelial proteins are key players mediating the stabilizing interactions and the adhesion to the cells lining the vascular lumen [103,224,225]. Recently, it was suggested that VlsE also promotes transient binding to the vasculature under flow via binding to dermatan sulphate and that a complex temporal choreography of P66, DbpA/B and OspC is required for the escape process from postcapillary venules [226,227]. Blood circulation is not the only mechanical stress that *B. burgdorferi* needs to overcome in order to infect the target tissues. During the vascular extravasation and after reaching the extracellular space, the bacterium faces a milieu of much lower shear stress than is experienced in the vasculature. Here, however, the pathogen needs to able to penetrate through dense and highly viscous structures with pore sizes much smaller than the diameter of borrelial cell. This is allowed by forming transiently stable interactions that enable pushing against the surrounding structures [104]. It was shown that DbpA and DbpB are critical borrelial surface molecules that allow for efficient dissemination of *Borrelia* in low shear stress and dense environments such as the extracellular matrix [13].

In contrast to pathogens that circulate only passively in the vasculature of a host, *B. burgdorferi* can actively influence its dissemination in the host in order to infect various organs and tissues. Multiple studies have shown that motility of *B. burgdorferi* is a critical virulence determinant and absolutely vital for pathogenesis throughout the enzootic cycle and the loss of motility leads to a non-infectious or attenuated phenotype [228–230]. The bacterium is able to adopt distinct motility states and transition between them as the pathogen colonizes the host [104]. The unique flat wave rotational movement of *Borrelia* is generated by 7–11 flagella located beneath the outer membrane in the periplasmic space. Since borrelial flagella do not extend outwards from the bacterial surface, *B. burgdorferi* is able to migrate with ease in highly viscous media, in which most other bacteria are significantly decelerated [231]. Therefore, the spirochaetes like to hide in the extracellular structures, making them less subject to circulating leukocytes. *Borrelia* with a mutation in the major flagellin gene *flaB* that did not synthesize flagella, was shown to be non-motile and rod-shaped [232]. As such, the flagella are arguably one of, if not the most critical virulence factors in the LD infection. Additionally, *B. burgdorferi* possesses an unusual peptidoglycan cell wall which significantly contributes to structural and morphological integrity of the bacterium and allows the spirochaete to withstand the high torque generated by the periplasmic flagella during borrelial translocation [233]. BpiP protein aids in sustaining the cellular integrity cell by binding the peptidoglycan wall and it influences the borrelial virulence in the host [234]. Notably, peptidoglycan was shown to be a persistent immunogen contributing to inflammation during acute infection and post-Lyme arthritis [46].

### Localization into specific niches in extracellular matrix

In contrast to RF *Borrelia*, LD *Borrelia* are rapidly cleared from the blood circulation and cannot multiply there [235]. When the adaptive immune responses of the host are fully activated, *B. burgdorferi* disappears from the bloodstream and disseminates to the organs, collagenous tissues, joints, and synovial fluid of the host [236]. The spirochaetes need to survive until they are transmitted back to competent ticks. At this time the successful persistence of the spirochaete within the host depends on evading the host’s immune system, rather than exploiting the host tissues for reproduction or growth [237]. The extracellular matrix provides an immune-privileged milieu for the pathogen [238]. The generally accepted theory is, after vascular extravasation, the spirochaetes colonize the ECM of multiple tissues and organs, which contain molecules that can be bound by *Borrelia*. These are commonly proteoglycans, biomolecules composed of a core protein together with long and negatively charged polysaccharide chains called glycosaminoglycans (GAGs). For example in joints, ECM of the articular cartilage contains a variety of GAGs such as chondroitin sulphates, keratan sulphate, dermatan sulphate as well as multiple collagen types [239,240]. LD *Borrelia* bind to different proteoglycans, which promotes tissue colonization and bacterial attachment to the cells [211,213,241,242]. The binding interactions with the host ECM components are mediated by outer surface molecules of *Borrelia* (Table 1). *B. burgdorferi* displays a dizzying diversity of abundant surface lipoproteins. At least 120 proteins that possess a lipid moiety are encoded in the borrelial genome [261], which represent nearly 8% of all borrelial open reading frames [262]. Out of 125 lipoproteins, 86 of these were shown to be secreted to the outer surface [261].

**Table 1.** Surface proteins of *B. burgdorferi* which are known to mediate attachment to the extracellular matrix (ECM) components of the vertebrate host.

ECM component	Protein	Reference
Collagen	CspA, BBA33	[243, 244]
Decorin	DbpA, DbpB	[245, 246]
Fibronectin	BBK32, RevA, RevB, BB0347, OspC	[205, 242, 247–249]
Integrins	BBK32, P66, BB0172, BBB07	[250–253]
Laminin	BmpA, ErpX, BB0406, CspA	[,^256^]
Glycosaminoglycans	DbpA, DbpB, BBK32, Bgp, OspC, Lmp1, VlsE	[205, 226, 257–260]

DbpA and DbpB exhibit binding activity toward various components of the ECM, including the proteins decorin, biglycan and various GAG molecules such as dermatan sulphate or heparin [108,211,241,245,263]. Fibronectin-binding activity was demonstrated for at least four outer membrane proteins, BBK32, RevA, RevB, and BB0347 [242,247–249]. BBK32 mutants display a significant defect in infectivity in the mouse infection studies and highlight the fundamental role of the protein in *B. burgdorferi* pathogenesis [248,264,265]. Similarly to BBK32, the lack of RevA significantly affects the pathogenesis in the mouse model of LD, although *revA* is not absolutely essential for infection [266]. The role of RevB and BB0347 in pathogenesis remains to be elucidated. BmpA, ErpX and BB0406 were shown to interact with laminin [254–256]. *B. burgdorferi* produces also collagen-binding proteins such as CspA and BBA33 [243,244] and it is able to bind several types of host integrins such as α3β1 integrin using P66, BB0172, and BBB07 proteins [250–253].

## Alternative pathogenic mechanisms

**Figure 5.**
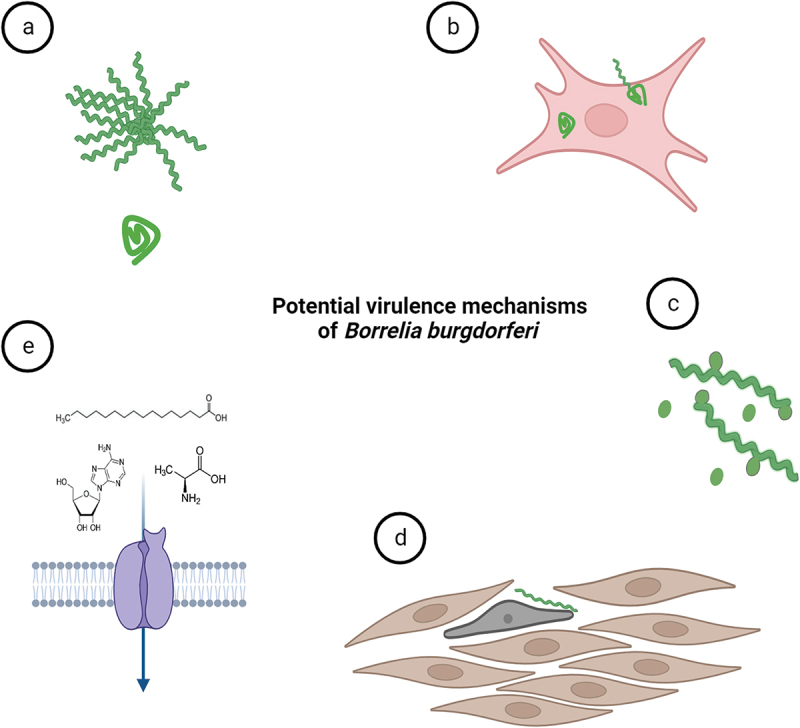
**Potential virulence mechanisms of *B. burgdorferi***. (a) *B. burgdorferi* can change its morphology as a response to environmental stress. Pleomorphic forms of *B. burgdorferi* such as biofilm-like structures (or aggregates) and round bodies could possibly help the spirochaetes to overcome prolonged stress conditions exerted for instance by antibiotics. (b) Although *B. burgdorferi* is considered extracellular parasite, the spirochete is occasionally found inside the nonphagocytic cells. The intracellular niche might help them to hide from immune responses. (c) outer membrane vesicles produced by the bacterium may modulate host immune responses. (d) structural transformation in cell shape and actin cytoskeleton of human cells were shown upon contact with *Borrelia*. (e) *B. burgdorferi* relies on uptake of essential nutrients such as amino acids, fatty acids and nucleosides from its host environments for survival and infection. Nutritional virulence might constitute important virulence factors for *B*. *burgdorferi.*

With the rise of modern research technologies and inflow of new studies, novel virulence mechanisms that might be exploited by the LD pathogen have come to light. These novel virulence activities are still either not fully accepted by the expert borrelial community or have not been thoroughly investigated yet. Confirmation of any of the purported virulence mechanisms (Figure 5) would significantly expedite our comprehension of *B. burgdorferi* infection, providing valuable insights to leverage in the creation of novel vaccines and other countermeasures. The pattern of the disease can be resolved into several discrete stages – host invasion, dissemination, colonization, and persistence. Interestingly, the discussed novel virulence mechanisms bear almost exclusively on the persistent stage of the disease.

### Structural variants of *B.* *burgdorferi*

Whereas no significant debate surrounds the issue of *B. burgdorferi* dissemination pathways within the host, the strategy for persistence inside the host is a different story. Under optimal conditions, *B. burgdorferi* has the typical flat wave morphology. The bacterium adopts the spiral form during multiple phases of its enzootic cycle – migration from the feeding tick midgut to the haemocoel and salivary glands, deposition in the skin, haematogenous dissemination, adhesion to and transmigration through the endothelium, and establishment of infection in distal niches, as it was evidenced by live and intravital imaging [12,14,105,267].

In the face of the hostile microenvironment conditions, the situation around the morphology of *Borrelia* becomes less clear. It was shown *in vitro* that *B. burgdorferi* can alter its cell shape into a different pleomorphic form under physiological stress, immune pressure, or antibiotic treatment [17,19,268–271], even inside tick midgut [272]. The morphologic variants, usually spherical and non-motile forms, of the *B. burgdorferi* are known under various terms in the literature. The terms such as L-forms, cystic forms, spheroplasts, or round bodies are commonly used. It was shown that the round bodies retain their flagella in the periplasmic space, have an intact and flexible cell envelope, and are able to dynamically transition back into the motile spirochaete under favourable conditions *in vitro* [17,271]. In addition, it was determined that the round bodies display lower metabolic activity and present different protein profiles and antigenicity than the spiral form [17,268].

The morphologic variants are often suggested to be associated with chronic or persistent LD and PTLDS, as they may enhance survival in hostile environmental conditions [18]. Chronic LD describes the cluster of symptoms that start after getting LD, persist despite antibiotic therapy and that result into long-term sequelae of infection. Although a systematic review of *B. burgdorferi* pleomorphs does not reveal a clear role in chronic LD [273], it was demonstrated that LD patients might have more intense responses to borrelial circular forms in comparison to spiral forms of *Borrelia*. These results suggest that round bodies might play a certain role in LD pathogenesis [268]. Using an *ex-vivo* murine skin model, it was observed that *Borrelia* can also form biofilm-like colonies made by spiral-shaped bacteria [274]. Biofilm represents an alternative lifestyle in which the microbes grow as structrured aggregates and adopt a multicellular behaviour that facilitates their survival under unfavourable conditions. The bacteria are typically held together by self-produced polymer matrixes. The presence of alginate-like polysaccharide was revealed in a study that explored the *Borrelia* aggregates *in vitro* [275].

Taken together, it is evident that the spiral, highly motile form is absolutely required for borrelial dissemination and colonization of the tick vector and the vertebrate host. We know that the motility states are dynamic as the spirochaetes were observed to transition between them in the dermis of the host [104] and the midgut of the tick [12]. It is also apparent that *B. burgdorferi* is exposed to many hostile environments and antimicrobial molecules during the pathogenic cycle and that the importance of morphologic variants for survival in hostile conditions should be considered in future research. Further studies need to be done to assess the potential contribution of morphologic and motility variants to virulence of *B. burgdorferi*.

### Intracellular niche

The ability of *B. burgdorferi* to maintain themselves for extended periods of time in the vertebrate hosts is critical for continuation of their enzootic cycle. The spirochaete can persist in multiple tissue sites despite strong immune pressure. Since *B. burgdorferi* is known to be an extracellular pathogen, the ECM is considered to be the ultimate protective niche for the spirochaete, where the organism is sequestered from the immune responses [238]. Nonetheless, one of the questions that is still a matter of debate, is whether *B. burgdorferi* is truly an obligate extracellular pathogen, or if the spirochaete is capable of living, and possibly reproducing, inside the host cells, when the conditions outside the cells are not favourable (for instance due to presence of strong immunity or antibiotics). A number of studies reported the intracellular localization of the bacteria inside non-phagocytic cells, especially fibroblasts [276–278], but also in synovial cells [279], endothelial cells [280], or glial and neuronal cells [281]. On the other hand, a recent study was not able to confirm the intracellular niche in the non-phagocytic mammalian cells using advanced correlative imaging approach, but they proved the propensity of the spirochaete for extracellular surface structures [107]. The stimuli that drives the pathogen to internalize into the non-phagocytic cells has yet to be determined.

Studies on *B. burgdorferi* infected murine fibroblasts has also suggested cyst-like *Borrelia* located inside these cells to possibly aid in host immune evasion by harbouring the bacterium in an inactive state. When the friendly environment is restored, the spirochaetes can revert back to a motile spirochaetal form [270,271,276]. Notably, coiling phagocytosis, the preferential phagocytic mechanism for *B. burgdoferi*, has been demonstrated to occur less often with non-motile forms than motile spirochaetes [268], suggesting that different receptors are present in the bacterial membrane of spiral and non-spiral *Borrelia*. Moreover, intracellular spirochaetes were observed in a variety of shapes, while simultaneously avoiding lysosomal colocalization during the coculture [19]. The differences in antigenic expression between borrelial pleomorphs plausibly explain the ability of the bacterium to adapt to different milieus and survive in highly adverse environments [19].

### *Borrelia-*induced structural transformations of host cells

*Borrelia* is a pathogen that depends distinctly on its host to survive, since the spirochaete lacks many metabolic pathways needed to produce its own nutrients. Recently, it has been suggested that *Borrelia* is able to hijack the molecular machinery of host cells to gain survival benefits [162,282], in a similar manner as was evidenced in many other human pathogens [283–285]. Metabolism generates energy as well as fundamental building blocks for every vital aspect of cell biology, including the formation of the cytoskeleton and extracellular matrix. Since many products of the cellular machinery are structural components of the cell and at the same time LD is characterized by tissue transformations of the host, it might be tempting to hypothesize that *Borrelia* directly affects the structural and mechanical properties of the host cells. Significant upregulation of genes coding for decorin or collagen type I was demonstrated in human fibroblasts upon cultivation with *B. burdorferi* species [278]. Induced alterations in cell shape and actin cytoskeleton of human cells were also confirmed upon contact with *Borrelia* [286]. The structural rearrangements of the host cells will likely be highly beneficial for the pathogen. For instance, the skeletal changes can provide a way for *Borrelia* to escape phagocytosis. Specifically, it was shown that uptake of *Borrelia* by macrophages is not a simple, one-directional process but rather a lengthy battle, where the back-and-forth movement of the spirochaete might create “tunnels” inside the macrophage [22]. To what extent is the pathogenesis caused by structural changes in the host organism is now a question worth pursuing as mechanical properties of the host cells might also be the critical determinant of the pathogen virulence [287].

Based on the intimate interactions that *Borrelia* form with the host cells, one can assume that the physical contact of *Borrelia* with the cell (probably with some of the outer surface virulent molecules of *Borrelia*) triggers the modulatory effects on cellular metabolism, which ultimately results in changes of cellular architecture and nanomechanical properties of the cell. In the literature, there are numerous studies showing that cells affected by a disease exhibit different nanomechanical properties [288] and that cell elasticity is a critical determinant of proper cell function [289]. Specifically in LD *Borrelia*, exposure of endothelial cells to spirochaetes led to decreased motility and physical forces in host cell monolayers and affected the mechanotransduction of the endothelial cells [25]. An understanding of the structural rearrangements and mechanical responses of human cells to pathogen infection may shed light on the origin of structure-related clinical manifestations such as skin fibrosis, morphea, or hyperkeratosis.

### Secretion to extracellular space

The capacity of bacteria to secrete proteins, polysaccharides, and various macromolecular membrane-derived complexes beyond the bacterial cell surface is often essential in the understanding of microbial pathogenesis. Frequently, bacterial pathogenicity depends fundamentally on the ability to secrete virulence-associated molecules which are located on the bacterial outer surface, released into the extracellular space or introduced directly into the host cell. Secretion systems have been thoroughly studied in a broad spectrum of Gram-negative species [290]. Contrary to the other bacterial systems, secretion of soluble proteins into the surrounding environment has been demonstrated rather sporadically in *Borrelia* due to limited secretory capabilities of spirochaetes [291].

Bacterial outer membrane vesicles (OMVs) have multifaceted roles as they can function as signal delivery vehicles for proteins, toxins, and other virulence factors, as well as genetic material and have the ability to modulate host immune responses [20,292]. OMVs are usually formed by two different mechanisms: by membrane blebbing or explosive cell lysis, with size between 10 and 300 nm and with a single membrane bilayer, which consist of almost the same outer membrane proteins as the membranes of the bacteria that the OMVs originated from [293,294]. Although the secretion of proteins to extracellular space is rare and largely uncharacterized in *Borrelia*, several studies have shown that LD spirochaetes are able to produce OMVs. *Borrelia* OMVs, also known as blebs in the literature, have been revealed to be released near the sites of cell division [295], and shedding can be observed both in culture [20] or *in vivo* inside tick midguts mostly at the outset of tick feeding [12]. The blebs were shown to be likely a transitional stage between the spiral and the round bodies form, with an expanded outer envelope and with a folded protoplasmic cylinder inside [17].

OMVs from *B. burgdorferi* were demonstrated to be on average 33 nm in diameter and contained antigenic proteins OspA, OspC, p39, peptidoglycan, and neutrophil attracting protein NapA [296,297]. Another *B. burgdorferi* protein termed Oms28 was reported to be secreted from borrelial cells into extracellular environment [298] and associated with borrelial OMVs [299]. GAG-binding outer surface membrane protein Bgp has been shown to be secreted from the borrelial cell as well [298,300]. The spirochaetes produce OMVs that contain double stranded DNA [296,301], which could presumably be a mechanism of horizontal gene transfer. Through the release of OMVs, *B. burgdorferi* can also transfer lipids and glycolipids to host epithelial cells. This lipid exchange could be an important process leading to antigen presentation and immune recognition that contributes to the pathogenesis of LD [302]. Nonetheless, the precise roles of OMVs in the pathogenesis of *Borrelia* infections remain to be defined.

### Nutritional virulence of LD *Borrelia*

Although LD spirochaetes are not known to significantly affect the host by directly competing for scarce nutrients with the host cells, the term “nutritional virulence” has been recently mentioned in connection with borrelial virulence [21,303]. Like many other obligate parasites, LD *Borrelia* species have significantly reduced their metabolic and biosynthetic capabilities. As a consequence, *B. burgdorferi* lacks genes encoding enzymes necessary for nucleic acid, amino acid, and fatty acid biosynthesis. The survival of the spirochaete in nature is thus fully dependent on salvage of essential nutrients from their vertebrate hosts and tick vectors. *B*. *burgdorferi* requires uptake of numerous components, such as cholesterol and long chain saturated and unsaturated fatty acids [302,304], purines [305], transition metals [164], and various carbon sources [306,307]. Several lines of evidence indicate that factors involved in nutrient acquisition might constitute key virulence determinants for *B*. *burgdorferi* [8]. *Borrelia* utilizes various scavenging strategies, obtaining macromolecules from the microenvironment and breaking them down to produce substrates for their own anabolic processes. Typically, the bacterium utilizes highly selective/unspecific porins (P66, P13, Oms28, BB0406) that open and close in response to specific stimuli and allow passive diffusion, or, active transport, which exploit the free energy from ATP hydrolysis to pump the solutes against concentration gradients [256,308,309].

Owing to its parasitic lifestyle, *B. burgdorferi* is restricted to utilizing only the nutrients that are available in its microenvironment. It is probable that the ability to exploit a variety of available carbohydrate sources during the enzootic cycle is essential for the survival of *B. burgdorferi* [307]. It has been reported that *B. burgdorferi* can utilize at least seven carbohydrates as the carbon source – glucose, mannose, N-acetylglucosamine, chitobiose, maltose, trehalose, and glycerol [310,311]. When multiple nutrient sources are available, the spirochaete likely takes advantage of the rapidly absorbable and high energy carbon sources, typically glucose. During the off-host starvation periods in the tick, the spirochaetes were shown to use glycerol as a carbohydrate source for glycolysis to maintain maximal *Borrelia* fitness [306]. A common regulatory mechanism in bacteria that represses genes associated with the utilization of secondary carbon sources has been coined carbon catabolite repression (CCR) [312]. There is no evidence of simple CCR in *B. burgdorferi* [15], rather the switch in carbon usage is orchestrated by mechanisms including various regulatory elements [313].

*B. burgdorferi* is unable to synthesize fatty acids *de novo*, elongate fatty acid side chains or process exogenous fatty acids via beta-oxidation [13]. Another noteworthy aspect is that *B. burgdorferi* differs from other bacteria and spirochaetes in that it mostly contains only two types of phospholipids in the outer and cytoplasmic membranes, phosphatidylglycerol and phosphatidylcholine [14]. In order to obtain exogenous choline and fatty acids, the spirochaete utilizes lipases to break down the lipids of the host [314]. Using the phosphatidylcholine synthase proteins, the spirochaete then synthesizes new lipids using the building blocks acquired from the host [315]. The lipolytic activity of multiple borrelial enzymes was shown to contribute to optimal infectivity of the bacterium [21,314], supporting fatty acid salvage as a virulence mechanism of *B. burgdorferi*. In addition, fatty acids are not only fundamental components of borrelial membrane bilayers, but these moieties are part of many virulence-associated, surface-exposed lipoproteins, needed for optimal infectivity during tick and host colonization. Osp-, Csp-, and Erp- lipoprotein families, DbpA/B, RevA/B, and VlsE belong among the well-known, surface lipoproteins tightly associated with borrelial pathogenic potential [261]. Palmitic acid is the most common fatty acid found in the lipoproteins of *B. burgdorferi* [316]. The interconnection of glycerol metabolism and glycolysis with lipoprotein biosynthesis have been found to depend on a function of two dehydrogenases, glycerol-3-phosphate dehydrogenase (GlpD) and glycerol-3-phosphate dehydrogenase (GpsA) [317]. GpsA is required for survival during nutrient stress and for mammalian infection [317]. The spirochaete is unable of *de novo* synthesis of NAD^+^ and therefore the molecule has to be recycled through the salvage pathways of nicotinamide. The enzyme that catalyzes the reaction (PncA) is a key element for mammalian infection [318].

## Conclusion

An understanding of the key aspects in the pathogenesis of LD is decisive to the effective care of patients affected with the disease. The virulence strategies of the infectious microbes are constantly evolving to maintain their ability to infect, disseminate, and proliferate in the host, which, in turn, forces the researchers to evolve new ways to detect and combat the pathogen. In this review, we have summarized the critical findings regarding the well-defined virulence factors of *B. burgdorferi* and potential novel virulence mechanisms that are being investigated under the scope of current research. Although much has been explored about the genes and proteins that play roles in disease development [8], the need for further extensive research is obvious to completely characterize the different factors underlying the infection caused by *B. burgdorferi*. The recent improvements in high-end technologies, which allow for instance whole-genome sequencing and enable much better resolution in microscopy and other structural biology techniques such as NMR, has directly benefitted the understanding of various aspects of LD, including borrelial phylogeny, virulence mechanisms, subversion of immune system, or host association of different *Borrelia* species. In this regard, especially targeted mutagenesis and complementation are key methods for studying genes of unknown function among bacterial pathogens including the LD spirochaete. In addition, genome sequencing of multiple *B. burdorferi* genospecies should significantly accelerate the knowledge on this bacterial complex. Until now, most of the data about borrelial virulence determinants come from genetic experiments performed on North American strains of a single species, *B. burgdorferi* ss [9]. Recent whole-genome sequences of several European and US originated species/strains makes adaptation and use of genetic techniques feasible in studying inherent differences between them [16,319–321].

The complexity of the clinical picture of LD requires a reliable diagnosis and a wide variety of pathologies. In case of untreated infection or absence of antibiotic therapy, disseminated spirochaetes can persist in the patient for many months while evading immune responses. Antibiotic treatment at the early stage of the disease is the only effective measure to clear the infection as no vaccine for human use is currently available. There are a few novel promising vaccine candidates in the development or exploratory stage [4,7]. In addition, the direct anti-*Borrelia* measures can be coupled with anti-tick strategies to deliver a whole new level of human protection [322]. Full characterization of the drivers involved in the pathogenic processes, understanding of the regulatory mechanisms, and further molecular and genetic analysis, will enable us to design better control and diagnostic approaches.

We aimed at highlighting a lot of the advances made in understanding the various virulence determinants of *B. burgdorferi*. It has also brought into sharper focus how much data we still need, to obtain a more in depth understanding of the virulence mechanisms that have been identified and those yet to be identified. The STM analysis was a major step toward this goal but needs to be followed through at the individual level for observing the virulence phenotype associated with each of the STM mutants. It is abundantly clear that to be able to obtain better drug-related interventions for patients, who suffer because of misdiagnosis or belated treatment, use of faster genetic methods/’omics’ studies is important. The uniqueness/plasticity of the genomes of the various *Borrelia* genospecies is the biggest challenge we face in obtaining a broader spectrum of medical/diagnostic options to counter this disease-causing bacterial pathogen.

## Data Availability

Data sharing is not applicable to this article as no datasets were generated or analysed in the current study.
